# The Defective Allele of *Aldehyde Dehydrogenase 2* Gene is Associated with Favorable Postoperative Prognosis in Hepatocellular Carcinoma

**DOI:** 10.7150/jca.33221

**Published:** 2019-10-03

**Authors:** Po-Han Huang, Ching-Chih Hu, Cheng-Hung Chien, Li-Wei Chen, Rong-Nan Chien, Yi-Shiuan Lin, Mei Chao, Chih-Lang Lin, Chau-Ting Yeh

**Affiliations:** 1 Liver Research Center, Chang Gung Memorial Hospital, Taoyuan, Taiwan; 2 College of Medicine, Chang Gung University, Taoyuan, Taiwan; 3 Liver Research Unit, Keelung Chang Gung Memorial Hospital, Keelung, Taiwan.; 4 Community Medicine Research Center, Keelung Chang Gung Memorial Hospital, Keelung, Taiwan.; 5 Wesley Girls High School, Taipei, Taiwan; 6 Department of Microbiology and Immunology, Chang Gung University, Taoyuan, Taiwan; 7 Division of Microbiology, Graduate Institute of Biomedical Sciences, Chang Gung University, Taoyuan, Taiwan

**Keywords:** *ALDH2* genotype, hepatocellular carcinoma (HCC), overall survival.

## Abstract

**Background:** The *Aldehyde dehydrogenase 2 (ALDH2)* mutant genotypes contain an allele encoding defective ALDH2 with reduced efficacy of alcohol metabolism leading to accumulation of highly toxic and carcinogenic acetaldehyde. It can induce unpleasant “Asian flush syndrome” and associate with increased risk of cancers. However, to date, little is known about *ALDH2* genotypes in relation to the postoperative prognosis of hepatocellular carcinoma (HCC).

**Methods:** From 2002 to 2012, 419 HCC patients receiving surgical resection of HCC were enrolled for *ALDH2-*rs671 genotyping and outcome correlation.

**Results:** Of the patients included, 202 were *ALDH2*-rs671 “GG” (wild type) and 217 were mutant (defective) “AA” + “GA” genotype. Kaplan-Meier analysis indicated that “GG” genotype significantly associated with shorter metastasis-free (P = 0.034) and overall (P = 0.005) survival, but not recurrence-free survival (P = 0.281). Univariate followed by multivariate Cox proportional hazard analysis showed that “GG” genotype was an independent clinical predictor for shorter time-to-distant metastasis (adjusted P = 0.019) and shorter overall survival (adjusted P = 0.001). Subgroup analysis showed that in patients with negative hepatitis B surface antigen, Edmonson's histology grade < 3, and aspartate transaminase > alanine transaminase, the *ALDH*2-rs671-GG genotype was associated with both shorter time-to-metastasis and shorter overall survival.

**Conclusions:** HCC patients carrying a defective allele of *ALDH2* had a favorable postoperative outcome.

## Introduction

Hepatocellular carcinoma (HCC), the major histological subtype of primary liver cancer, ranks the fifth most commonly diagnosed solid cancer in men and the seventh in women as well as the third leading cause of cancer-related death worldwide 1. HCC is a multifactorial disease. Hepatitis B virus, hepatitis C virus, and alcohol consumption have been considered the three most important etiologic factors for HCC 2-5. There are several potentially curative treatments for early-stage HCC, including surgical resection, radiofrequency ablation therapies, and liver transplantation 6-9. Patients treated at an early stage can usually achieve complete remission. However, a large proportion of them suffers from subsequent cancer recurrence and distant metastasis, of whom HCC often progresses rapidly into intermediate or advanced stages. For patients who are diagnosed at unresectable stages, palliative treatments are recommended, including transcatheter arterial chemoembolization, radiotherapy, chemotherapy, targeted therapy, and immune therapy 10-12. Although the therapeutic outcome has improved remarkably in recent years, the prognosis of advanced HCC remains grave. Therefore, it is pivotal to identify postoperative prognosis factors so that researchers could devise novel adjuvant treatments to reduce the recurrence rate in selected groups of patients.

Alcohol is oxidized to acetaldehyde by alcohol dehydrogenase, which in turn is oxidized to acetate by aldehyde dehydrogenase. The *aldehyde dehydrogenase 2* gene (*ALDH2*), encodes a mitochondrial enzyme, which is the key enzyme responsible for alcohol metabolism. It contains two alternative alleles (rs617 - “G” or “A”) correlated with differential efficacy of alcohol metabolism 13, 14. Individuals heterozygous or homozygous for the lysine encoding (named “A” or *2) allele at the single nucleotide polymorphism (SNP) glu504lys (rs671) of *ALDH2* have an enzyme possessing greatly reduced ability to metabolize acetaldehyde, which can reach to as low as 17-50% of the wild type. These individuals accumulate acetaldehyde when consuming alcohol, suffer from alcohol intolerance symptoms, but therefore carry a decreased risk for alcohol dependence 15. Of note is that about 40% of the Eastern Asian populations carry the mutation phenotype 16. Additionally, it is well known that acetaldehyde, rather than ethanol, is highly toxic, carcinogenic, and mutagenic, and has been determined as the cause of “Asian flush syndrome” - unpleasant symptoms after alcohol intake with nausea, facial flushing, muscle weakness, tachycardia, palpitation, perspiration, headache, and sleepiness 17. Furthermore, a recent review proposed that the differential ALDH2 expression may dedicate to a wide variety of human diseases, including cardiovascular diseases, diabetes, and cancers 18. However, the role of *ALDH2* genotype in the disease progression and the postoperative prognosis of HCC remains largely unstudied.

In the present study, we aimed to investigate whether the *ALDH2* genotype associated with the postoperative outcome of HCC.

## Materials and Methods

### Patients

This was a retrospective cohort study approved by the institutional review board, Chang Gung Medical Center, Taoyuan, Taiwan. In total, 419 consecutive HCC patients receiving surgical resection of liver tumors from 2002 to 2012 with available liver tissues in Chang Gung Medical Center, Taoyuan, Taiwan, were included. At our institute, all HCC patients must be evaluated before and after surgery to ensure that a clean margin of more than 1 cm was obtained. More importantly, the diagnosis of HCC was confirmed by the pathological results of surgical specimens. Therefore, the inclusion criteria included pathological diagnosis of HCC, curative resection, no anticancer therapy received before the surgery, complete clinicopathological data, regular follow‑up, and reliable medical records. The exclusion criteria included pregnancy, questionable pathological diagnosis of HCC, and other co‑existing malignancies prior to HCC resection. All samples were frozen to - 70° C immediately after surgical operation and stored in Tissue Bank, Chang Gung Medical Center until used. The clinicopathological data were retrospectively reviewed, including gender, age, HBV surface antigen (HBsAg), antibody against HCV (anti-HCV), alcoholism, liver cirrhosis status, presence of ascites on surgery, Edmonson's histology grading, microvascular invasion, macrovascular invasion, presence of tumor capsule, number of tumor, largest tumor size, alpha-fetoprotein (AFP), albumin, bilirubin, prothrombin time, creatinine, aspartate aminotransferase (AST), alanine aminotransferase (ALT), date of surgical resection, date of tumor recurrence or metastasis, and date of last follow-up or HCC related death.

Alcoholism in this study was defined as documented daily alcohol consumption > 40 g/day for males or > 20 g/day for female, over a period of > 10 years, combined with psychological and physical dependence.

### *ALDH2* genotyping

Genotyping of the *ALDH2*-rs671 SNP was performed on genomic DNA extracted from the liver tissues (para-neoplastic, non-cancerous parts) using QIAamp DNA Mini kits (Qiagen, Düsseldorf, Germany). Primers were designed to amplify the region of *ALDH2* gene containing the variant rs671. To ensure that specific amplicons were correctly amplified, nested PCR was carried out by using primers: (a) 5′- TAAAGACTTTGGGGCAATACAGG -3′, (b) 5′- CCCAGCAAATGACCGCATA -3′, (c) 5′- AAGAGTGATTTCTGCAATCTCG -3′, and (d) 5′-CCTCAGTATTTCTCATGGGAC -3′. The first amplification reaction mixture contained DNA (5 µl), the primers (a) and (b) (10 µM; 0.25 µl each), the Taq DNA pol 2.0 master mix red (containing 1.5 mM MgCl_2_; 25 µl; Amplicon, Glostrup, Denmark) and double-distilled water (19.5 µl). Temperature for the PCR procedure was set at 95° C for an initial denaturation of 5 min, followed by 35 cycles of denaturation-annealing-extension procedure at 94° C, 58^0^ C and 72° C, respectively, holding denaturation and annealing temperature for 1 min and extension temperature for 3 min in a cycle. The procedure was then followed by a final extension with a temperature of 72°C for 10 min. Then, 5 μl of the first PCR products were used for amplification with the nested primers (c) and (d) for 35 cycles (94°C for 1 minute, 55°C for 1 minute, and 72°C for 1 minute). An aliquot of water was included for parallel PCR amplification in each batch, as a negative control. Finally, conventional Sanger sequencing was performed for direct sequence reads of *ALDH2* genotype.

### Statistical analysis

Dichotomized data was expressed as numbers and ratios (%) and compared by use of Chi-square test or Fisher's exact tests, where appropriate. Parametric data was expressed as mean ± standard deviation and compared by use of two-sample t-test or the Mann-Whitney U-test, where appropriate. Overall survival was calculated from the date of surgery to the date of death or last follow-up. Time to recurrence or distant metastasis was calculated from the date of surgery to the date of recurrence or distant metastasis, respectively. Univariate and multivariate Cox proportional hazard models were used to estimate survivals for clinicopathological and genotypic variables. In this study, significant factors identified from univariate analysis were included for multivariate Cox proportional hazards. The Kaplan-Meier method was used to estimate the survival probability between the different genotype groups, and the log-rank test was used to compare the survivals. All tests were two-tailed, and a P < 0.05 was considered statistically significant. All statistical analyses were performed using Statistical Package for the Social Sciences (SPSS) statistics Version 20 (SPSS, Chicago, IL, USA).

## Results

### Basic characteristics of patients included

Of the 419 patients included, 202 were *ALDH2* “GG” genotype. Their basic clinicopathological and genotypic characteristics were summarized in Table 1. Notably, the etiological analysis showed 111 (26.5%) were alcoholism, 303 (72.3%) were HBsAg-positive, and 101 (24.1%) were anti-HCV-positive. Patients with more than one etiological factor were not uncommon.

### Comparison between HCC patients carrying ALDH2 GG and non-GG genotype

A detailed comparison of all clinicopathological features between HCC patients carrying *ALDH2* “GG” and “non-GG” (“AA” + “GA”) genotype was shown in Table 2. In summary, patients with the “GG” genotype were significantly associated with alcoholism (35.6% versus 18%; P < 0.001). No significant differences were observed for other clinicopathological variables. Strikingly, even under the strict definition of alcoholism (see Methods), there remained 18% of our patients with “non-GG” or mutant genotypes having alcoholism.

### *ALDH2* genotype was associated with distant metastasis and overall survival in HCC

**Figure 1:**
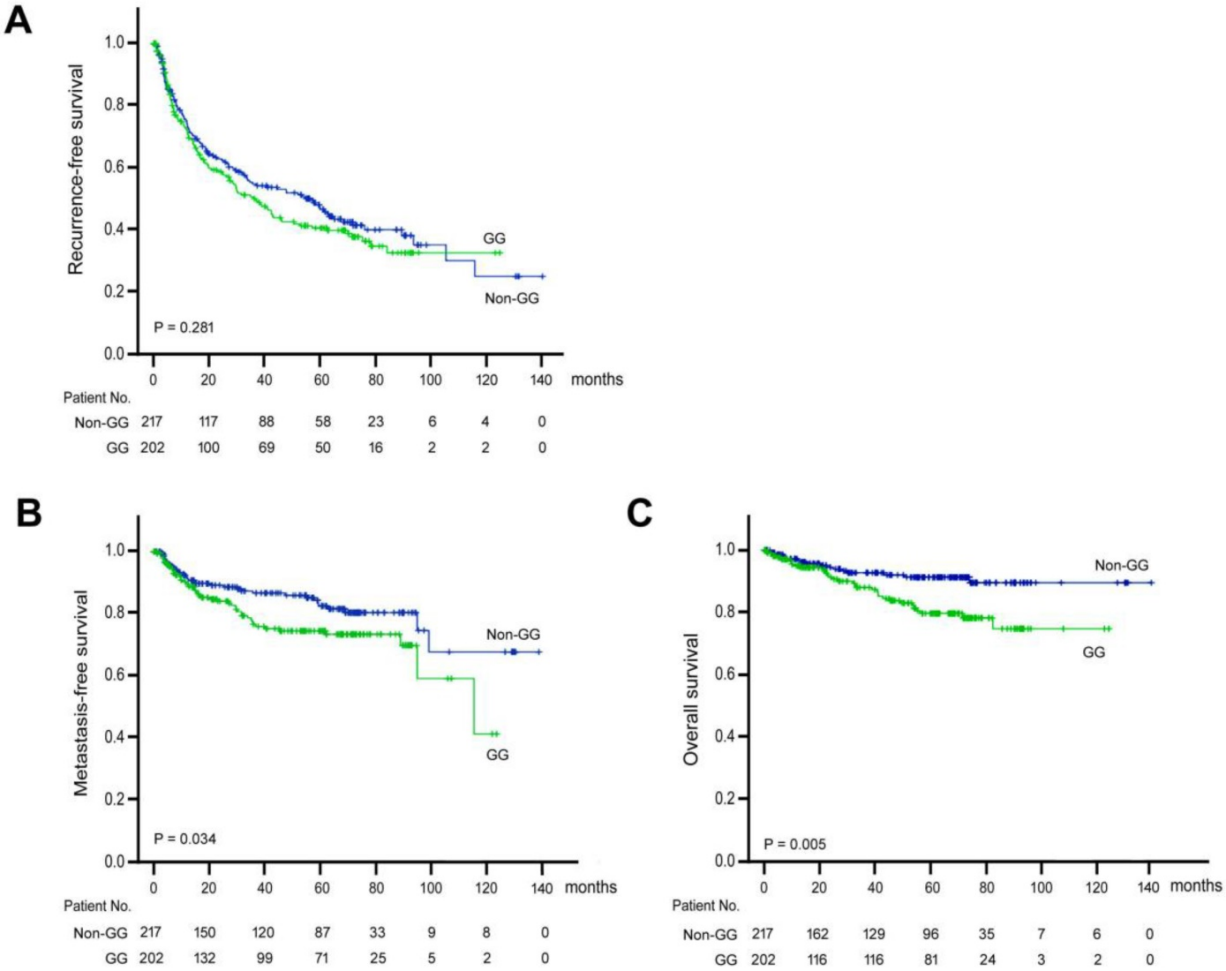
Kaplan-Meier analysis of postoperative outcomes in HCC patients carrying *ALDH2* “GG” versus “non-GG” genotype. (A) Recurrent-free survival. (B) Metastasis-free survival. (C) Overall survival. Green line, “GG” genotype; Blue line, “non-GG” genotype.

Kaplan-Meier analysis was performed to understand the prognostic significance of *ALDH2* genotype (Figure 1). It was discovered that the “GG” genotype was significantly associated with shorter metastasis-free (P = 0.034) and overall (P = 0.005) survival. However, the “GG” genotype was not associated with recurrence-free survival (P = 0.281).

Cox proportional hazard model was thus used to examine the association between clinical factors and time-to-distant metastasis after surgical resection of HCC (Table 3). Univariate analysis revealed that *ALDH2* “GG” genotype, microvascular invasion, macrovascular invasion, higher tumor number, larger tumor size, AFP, and AST were significantly associated with a shorter time-to-distant metastasis. After adjusted for other confounding factors, *ALDH2* “GG” genotype (P = 0.019), microvascular invasion (P < 0.001), macrovascular invasion (P = 0.006), larger tumor size (P = 0.017), and higher AFP (P = 0.003) remained as independent predictors for a shorter time-to-distant metastasis.

Similarly, Cox proportional hazard model was used to examine the association between clinical factors and overall survival after surgical resection of HCC (Table 4). Univariate analysis revealed that *ALDH2* “GG” genotype, presence of ascites on surgery, microvascular invasion, macrovascular invasion, higher tumor number, larger tumor size, albumin, and AST were significantly associated with shorter overall survival. After adjusted for other confounding factors,* ALDH2* “GG” genotype (P = 0.001), presence of ascites on surgery (P = 0.021), microvascular invasion (P = 0.011), and higher AST (P = 0.019) remained to be independent predictors for a shorter overall survival.

### Subgroup analysis to identify patient subgroups wherein *ALDH2* genotype effectively predicted postoperative outcomes

**Figure 2:**
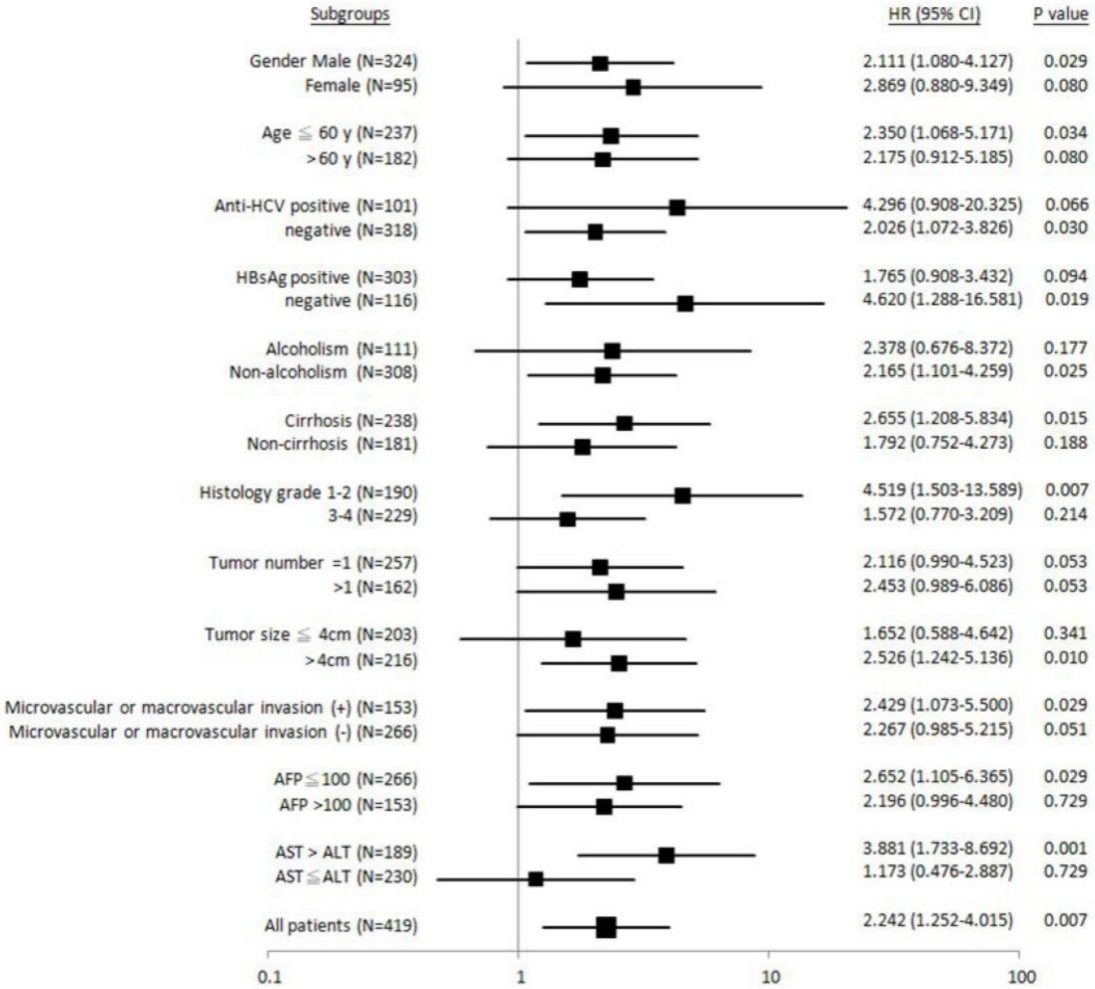
Forest plot analysis for overall survival in relation to *ALDH2* “GG” versus “non-GG” genotype in different clinical subgroups.

**Figure 3:**
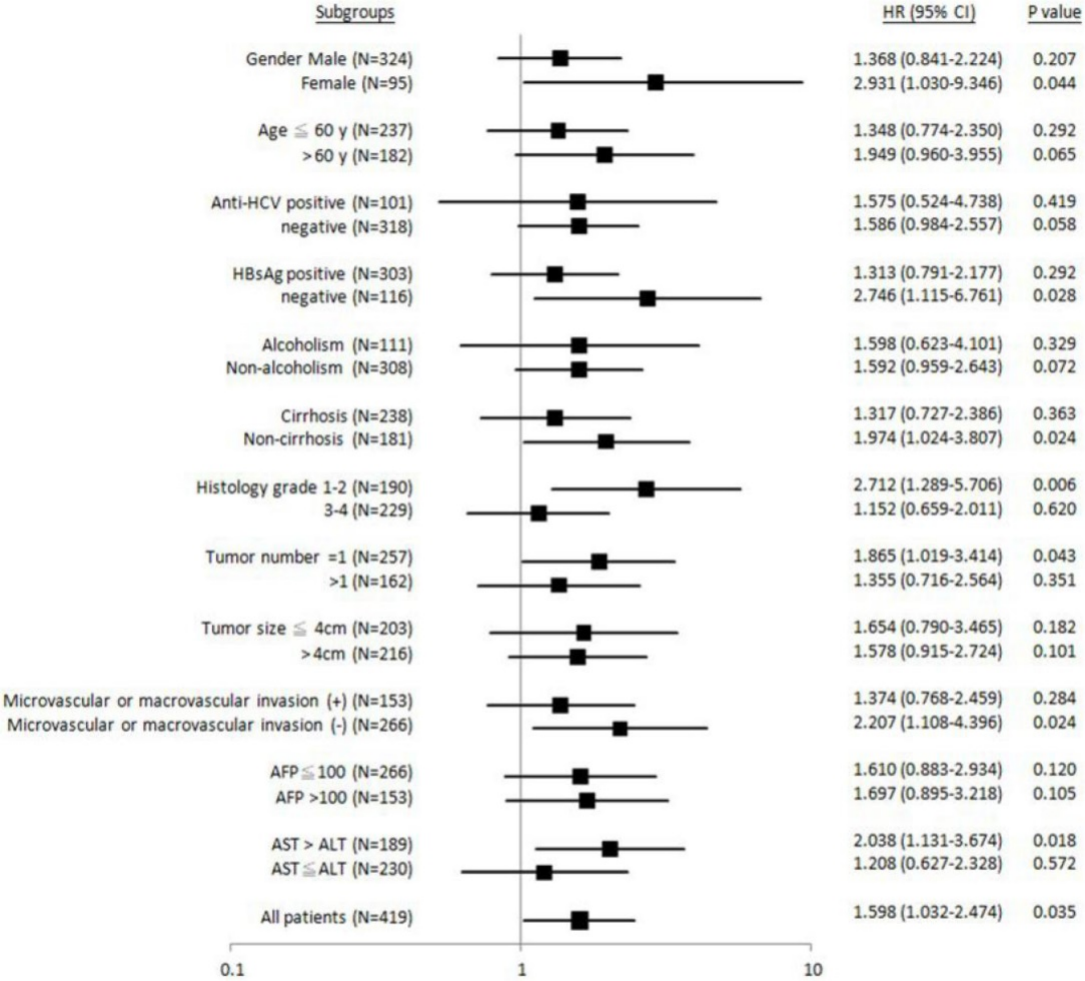
Forest plot analysis for metastasis-free survival in relation to *ALDH2* “GG” versus “non-GG” genotype in different clinical subgroups.

**Figure 4:**
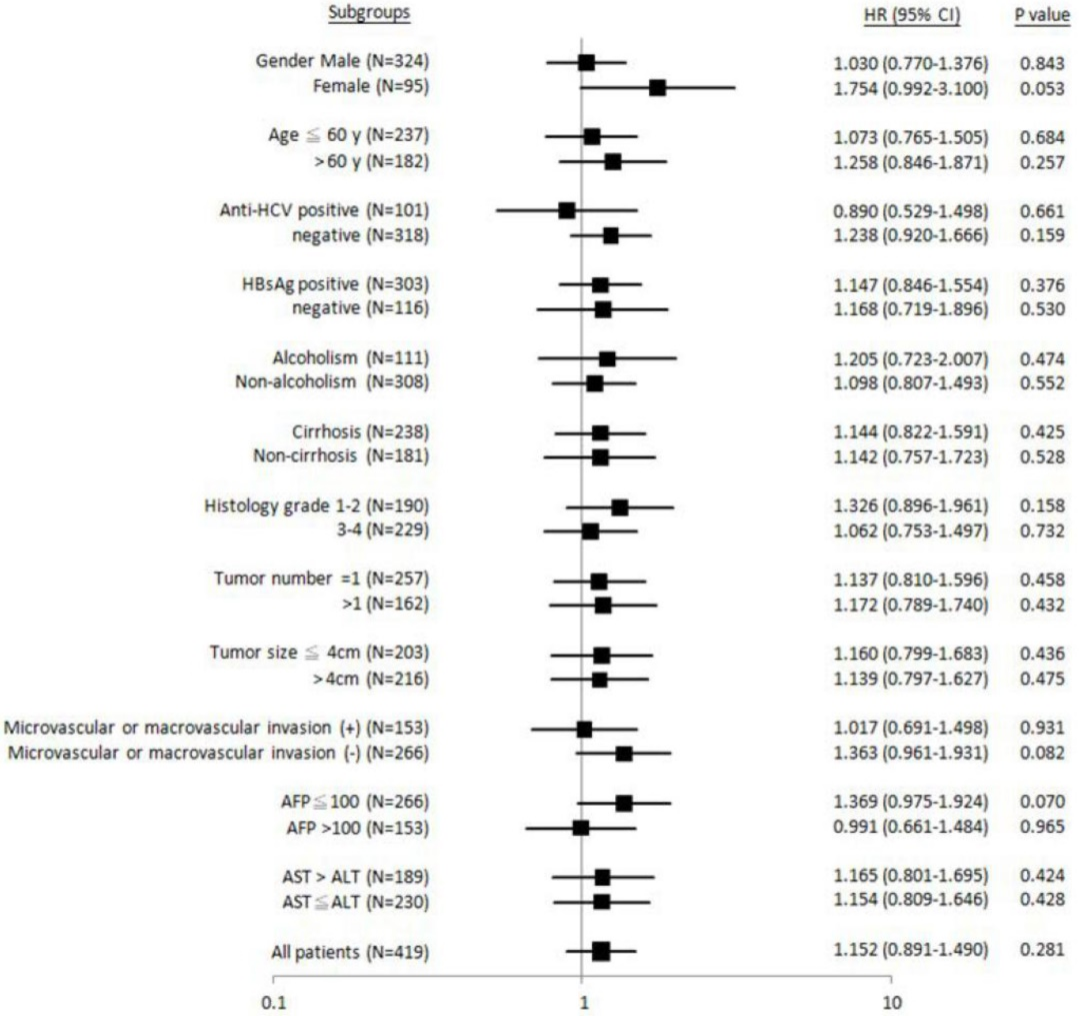
Forest plot analysis for recurrence-free survival in relation to *ALDH2* “GG” versus “non-GG” genotype in different clinical subgroups.

To gain more insight into why *ALDH2*-GG genotype was associated with unfavorable postoperative outcomes, we divided patients into different clinical subgroups for further analysis (Figure 2-4). It was found that patients with male gender, age £ 60 years old, anti-HCV-negative, HBsAg-negative, non-alcoholism, liver cirrhosis, Edmonson's histology grading 1-2, tumor size > 4 cm, presence of microvascular or macrovascular invasion, AFP £ 100 ng/mL, or AST > ALT, the “GG” genotype was significantly associated with shorter overall survival (Figure 2).

Similarly, it was found that patients with female gender, HBsAg-negative, non-liver cirrhosis, Edmonson's histology grading 1-2, tumor number = 1, absence of microvascular or macrovascular invasion, or AST > ALT, “GG” genotype was significantly associated with shorter metastasis-free (Figure 3). No subgroups were identified, where the “GG” genotype was associated with recurrence-free survival (Figure 4).

Taken together, in three subgroups: HBsAg-negative, Edmonson's histology grading 1-2, and AST > ALT, effective “GG” association with unfavorable postoperative outcomes was found in both time-to-metastasis and overall survival subgroup analysis.

## Discussion

In the present study, we aimed to investigate the association between *ALDH2* genotype and postoperative prognosis of HCC. Several interesting observations were made. Despite the low prevalence of alcoholism found in our HCC patients, the data still demonstrated that the wild genotype of *ALDH2*-“GG” was strongly associated with alcoholism, consistent with our current knowledge regarding ALDH2. Surprisingly, patients with *ALDH2*-“GG” genotype, who should have a more efficient alcohol metabolic enzyme, had significant shorter metastasis-free and overall survival, as compared with those with mutant genotypes *ALDH2*-“AA” or “GA”. We were initially puzzled by this seemingly contradictory result. However, after subgroup analysis was performed, it was found that in patients with HBsAg-negative, low histology grade and AST > ALT, the predictive value of ALDH2 genotpye was consistent for distant metastasis and overall survival. Patients with these characteristics were those having a heavy alcoholic intake, but not necessarily reaching alcoholism (with psychological and physical dependence). In Taiwan and many Asian countries, heavy alcohol drinking maybe needed on social occasions but not necessarily developed into a drinking habit. Most of these patients had AST > ALT and negative HBsAg. When diagnosed as HCC, patients with *ALDH2*-“AA” or “GA” genotype could easily abstain from the social-occasion-associated alcohol drinking (because of intolerance), whereas patients with *ALDH2*-“GG” were less likely to abstain themselves, partly because of established alcoholic addiction (alcoholism, see Table 2). As a result, *ALDH2*-“GG” was associated with an unfavorable prognosis.

Viral hepatitis plays a predominant role in the etiology and prognosis of HCC because of the high prevalence rate of chronic HBV or HCV infection in Asia 2, 4, 19. Fortunately, the prevalence of chronic HBV infection is declining due to the implementation of HBV vaccination programs 20. In addition, potent antiviral agents have been developed and widely applied for the treatment of viral hepatitis 21, 22. But as the number of alcoholic patients increases, the impact of alcohol and its metabolic genes on HCC development required more attention. In our patients included, the prevalence of HBV and HCV was high, 72.3% of HBsAg-positive and 24.1% of anti-HCV-positive. However, when Cox proportion hazard model was used to determine predictors for distant metastasis or overall survival, viral hepatitis and alcoholism were found to have no predictive effect. On the other hand, *ALDH2* “GG” genotype and AST elevation (suggesting heavy alcohol drinking but not necessarily alcoholism) were significantly outcome predictors. Out results argued that after HCC had developed, forced abstention from alcohol was needed for patients with* ALDH2* “GG” genotype.

Many previous studies have focused on the relationship between HCC occurrence and *ALDH2* genotype, and the results are inconsistent. The mutant genotype of *ALDH2* may have direct or indirect impacts to be implicated in the development of HCC, whether or not it is combined with viral hepatitis or alcohol ingestion 23, 24. On the other hand, some reports indicated that *ALDH2* mutant genotype may instead have protective effect, while *ALDH2* wild genotype serves as a predictor for HCC development 7, 25. However, there are still other reports suggested that *ALDH2* mutant genotype has only limited (not statistically significant) contribution for the risk of HCC 26, 27. To the best of our knowledge, the present study differs from previous studies as it is the first attempt to identify *ALDH2* genotype as an effective predictor for postoperative prognosis in HCC, but not as a risk factor for HCC development. Importantly, wild type ALDH2 can effectively metabolize alcohol and thus less likely to accumulate carcinogenic acetaldehyde. Therefore, one would expect that *ALDH2* wild type should be associated with a better postoperative prognosis. However, our data argued against this view. According to our subgroup analysis, we believed that both *ALDH2* genotypes and changes of drinking behavior after HCC resection were important determinants for postoperative prognosis (with the latter more important). As such, in patients receiving HCC surgical treatment, it is recommended to check the *ALDH2* genotype and to give alcohol abstinence order to those with wild type *ALDH2-“GG”*.

In order to further decipher our findings, we had conducted a small-scale validation study in which 87 adult patients were enrolled, who were diagnosed as HCC and received curative radiofrequency ablation therapies owing to unsuitable for operation (with more advanced stages of cirrhosis). Of the 87 patients included, 45 (51.7%) were *ALDH2*-“GG” (wild type). Compared to the “non-GG” mutant genotype, the HCC recurrence incidence of the “GG” genotype was borderline significantly higher (76.6% vs. 57.1%; P = 0.069). However, the “GG” genotype demonstrated no significantly difference for the incidence of distant metastasis and overall survival, albeit numerically higher (28.9% vs 23.8%; P = 0.591; 48.9% vs 45.2%; P = 0.733, respectively). Taken together, despite that these patients were in a more advanced stage of cirrhosis,* ALDH2*-“GG” (wild type) still associated with a poorer prognosis (higher recurrence).

In conclusion, we have discovered that *ALDH2* “GG” genotype is an independent predictor for shorter overall survival and metastasis-free survival in HCC patients treated by surgical resection.

## Figures and Tables

**Table 1:** Basic clinical data for HCC patients included.

Clinical parameters	
ALDH2 genotype, “GG”, n (%)	202 (48.2%)
Gender, male, n (%)	324 (77.3%)
Age, years, mean ± SD	56.0 ± 14.2
Anti-HCV, positive, n (%)	101 (24.1%)
HBsAg, positive, n (%)	303 (72.3%)
Alcoholism, yes, n (%)	111 (26.5%)
Cirrhosis, yes, n (%)	238 (56.8%)
Ascites, yes, n (%)	28 (6.7%)
Microvascular invasion, yes, n (%)	135 (32.2%)
Macrovascular invasion, yes, n (%)	43 (10.3%)
Histology grade	
1, n (%)	11 (2.6%)
2, n (%)	179 (42.7%)
3, n (%),	192 (45.8%)
> 3, n (%)	37 (8.8%)
Capsule, yes, n (%)	305 (72.8%)
Tumor number	
1, n (%)	227 (61.4%)
2, n (%)	94 (22.4%)
3, n (%)	47 (11.2%)
>3, n (%)	21 (5.0%)
Tumor size, cm, mean ± SD	5.8 ± 4.0
Alpha-fetoprotein, ng/mL, median (range)	25.0 (< 2 to 685353)
Albumin, g/L, mean ± SD	4.0 ± 0.6
Bilirubin, mg/dL, mean ± SD	1.1 ± 1.4
Prothrombin time, sec, mean ± SD	12.1 ± 1.4
Creatinine, mg/dL, mean ± SD	1.1 ± 0.9
AST, U/L, mean ± SD	65.2 ± 85.4
ALT, U/L, mean ± SD	66.5 ± 84.2

**Table 2:** Comparison of clinical parameters between HCC patients carrying ALDH2 “GG” and non-“GG” (“AA” + “GA”) genotype.

Clinical parameters	ALDH2 genotype		
	“GG” (n = 202)	“GA” + “AA” (n = 217)	P
Gender, male, n (%)	155 (76.7%)	169 (77.9%)	0.779
Age, years, mean ± SD	54.6 ± 14.4	57.2 ± 13.9	0.060
Anti-HCV, positive, n (%)	51 (25.2%)	50 (23.0%)	0.598
HBsAg, positive, n (%)	148 (73.3%)	155 (71.4%)	0.674
Alcoholism, yes, n (%)	72 (35.6%)	39 (18.0%)	**< 0.001**
Cirrhosis, yes, n (%)	115 (56.9%)	123 (56.7%)	0.959
Ascites, yes, n (%)	13 (6.4%)	15 (6.9%)	0.845
Microvascular invasion, yes, n (%)	56 (27.7%)	79 (36.4%)	0.057
Macrovascular invasion, yes, n (%)	43 (10.3%)	24 (11.1%)	0.577
Histology grade, > 2, n (%)	105 (52.0%)	124 (57.1%)	0.289
Capsule, yes, n (%)	148 (73.3%)	157 (72.4%)	0.833
Tumor number, > 1, n (%)	77 (38.1%)	85 (39.2%)	0.825
Tumor size, cm, mean ± SD	5.7 ± 4.0	5.8 ± 4.0	0.707
Alpha-fetoprotein, ng/mL, median (range)	25.0 (< 2 to 443209)	33.0 (< 2 to 685353)	0.490
Albumin, g/L, mean ± SD	4.0 ± 0.5	3.9 ± 0.6	0.134
Bilirubin, mg/dL, mean ± SD	1.1 ± 1.6	1.1 ± 1.3	0.792
Prothrombin time, sec, mean ± SD	12.1 ± 1.4	12.1 ± 1.3	0.764
Creatinine, mg/dL, mean ± SD	1.1 ± 1.1	1.1 ± 0.7	0.994
AST, U/L, mean ± SD	60.9 ± 70.0	69.1 ± 97.6	0.326
ALT, U/L, mean ± SD	59.2 ± 60.6	73.2 ± 101.1	0.089

**Table 3:** Cox proportional hazard analysis for association between clinical factors and time-to-distant metastasis.

Clinical parameters	Univariate analysis		Multivariate analysis	
	Hazard Ratio (95% CI)	P	Hazard Ratio (95% CI)	P
ALDH2 genotype, “GG” = 1	1.598 (1.032 - 2.474)	**0.035**	1.704 (1.091 - 2.661)	**0.019**
Gender, male =1	1.217 (0.713 - 2.075)	0.471		
Age, per year increase	0.991 (0.976 - 1.007)	0.266		
Anti-HCV, positive = 1	0.630 (0.355 - 1.120)	0.116		
HBsAg, positive = 1	1.011 (0.620 - 1.648)	0.966		
Alcoholism, yes = 1	1.063 (0.657 - 1.721)	0.803		
Cirrhosis, yes = 1	0.811 (0.527 - 1.249)	0.342		
Ascites, yes = 1	0.606 (0.191 - 1.922)	0.395		
Microvascular invasion, yes = 1	2.984 (1.938 - 4.595)	**< 0.001**	2.449 (1.568 - 3.824)	**< 0.001**
Macrovascular invasion, yes = 1	2.806 (1.623 - 4.852)	**< 0.001**	2.218 (1.256 - 3.916)	**0.006**
Histology grade, per grade increase	1.237 (0.907 - 1.687)	0.178		
Capsule, yes = 1	0.862 (0.539 - 1.378)	0.536		
Tumor number, per number increase	1.323 (1.094 - 1.601)	**0.004**	1.138 (0.926 - 1.397)	0.219
Tumor size, per cm increase	1.114 (1.068 - 1.161)	**< 0.001**	1.063 (1.011 - 1.118)	**0.017**
Alpha-fetoprotein, per 1000 ng/mL increase	1.005 (1.002 - 1.007)	**< 0.001**	1.004 (1.001 - 1.007)	**0.003**
Albumin, per g/L increase	0.830 (0.557 - 1.237)	0.361		
Bilirubin, per mg/dL increase	0.841 (0.571 - 1.238)	0.380		
Prothrombin time, per sec increase	1.016 (0.870 - 1.187)	0.839		
Creatinine, per mg/dL increase	0.592 (0.273 - 1.284)	0.184		
AST, per U/L increase	1.003 (1.001 - 1.005)	**0.012**	1.001 (0.998 - 1.005)	0.420
ALT, per U/L increase	0.999 (0.996 - 1.002)	0.501		

**Table 4:** Cox proportional hazard analysis for association between clinical factors and overall survival.

Clinical parameters	Univariate analysis		Multivariate analysis	
	Hazard Ratio (95% CI)	P	Hazard Ratio (95% CI)	P
ALDH2 genotype, “GG” = 1	2.242 (1.252 - 4.015)	**0.007**	2.751 (1.513 - 5.002)	**0.001**
Gender, male =1	0.952 (0.507 - 1.789)	0.879		
Age, per year increase	0.997 (0.978 - 1.017)	0.774		
Anti-HCV, positive = 1	0.738 (0.370 - 1.474)	0.390		
HBsAg, positive = 1	0.967 (0.522 - 1.790)	0.914		
Alcoholism, yes = 1	1.310 (0.725 - 2.369)	0.371		
Cirrhosis, yes = 1	0.927 (0.532 - 1.614)	0.788		
Ascites, yes = 1	2.961 (1.391 - 6.302)	**0.005**	2.560 (1.154 - 5.677)	**0.021**
Microvascular invasion, yes = 1	2.177 (1.253 - 3.784)	**0.006**	2.125 (1.191 - 3.794)	**0.011**
Macrovascular invasion, yes = 1	2.449 (1.189 - 5.042)	**0.015**	1.776 (0.831 - 3.797)	0.138
Histology grade, per grade increase	1.350 (0.913 - 1.996)	0.133		
Capsule, yes = 1	0.717 (0.401 - 1.284)	0.263		
Tumor number, per number increase	1.292 (1.018 - 1.639)	**0.035**	1.173 (0.904 - 1.521)	0.229
Tumor size, per cm increase	1.084 (1.024 - 1.148)	**0.006**	1.016 (0.954 - 1.082)	0.630
Alpha-fetoprotein, per 1000 ng/mL increase	1.002 (0.998 - 1.005)	0.333		
Albumin, per g/L increase	0.528 (0.332 - 0.841)	**0.007**	0.662 (0.390 - 1.122)	0.125
Bilirubin, per mg/dL increase	1.102 (0.973 - 1.249)	0.126		
Prothrombin time, per sec increase	1.024 (0.844 - 1.242)	0.809		
Creatinine, per mg/dL increase	0.937 (0.658 - 1.335)	0.720		
AST, per U/L increase	1.004 (1.002 - 1.006)	**0.001**	1.004 (1.001 - 1.007)	**0.019**
ALT, per U/L increase	1.001 (0.998 - 1.004)	0.504		

## Abbreviations

AFP: alpha-fetoprotein
ALDH2: aldehyde dehydrogenase 2 gene
ALT: alanine aminotransferase
anti-HCV: antibody against HCV
AST: aspartate aminotransferase
HBsAg: hepatitis B virus surface antigen
HCC: hepatocellular carcinoma
